# GLK/MAP4K3 overexpression associates with recurrence risk for non-small cell lung cancer

**DOI:** 10.18632/oncotarget.9410

**Published:** 2016-05-17

**Authors:** Chung-Ping Hsu, Huai-Chia Chuang, Ming-Ching Lee, Hsiao-Hui Tsou, Li-Wen Lee, Ju-Pi Li, Tse-Hua Tan

**Affiliations:** ^1^ Division of Thoracic Surgery, Department of Surgery, Taichung Veterans General Hospital, Taichung, 40705, Taiwan; ^2^ Faculty of Medicine, School of Medicine, National Yang-Ming University, Taipei, 11221, Taiwan; ^3^ Immunology Research Center, National Health Research Institutes, Zhunan, 35053, Taiwan; ^4^ Institute of Clinical Medicine, School of Medicine, National Yang-Ming University, Taipei, 11221, Taiwan; ^5^ Division of Biostatistics and Bioinformatics, Institute of Population Health Sciences, National Health Research Institutes, Zhunan, 35053, Taiwan; ^6^ Department of Pathology & Immunology, Baylor College of Medicine, Houston, Texas, 77030, USA

**Keywords:** MAP4K3, GCK-like kinase (GLK), cancer recurrence, NSCLC, lung cancer

## Abstract

Lung cancer is the leading cause of cancer death worldwide. Non-small cell lung cancer (NSCLC) accounts for 85% of total lung cancers; 40% to 60% of NSCLC patients die of cancer recurrence after cancer resection. Since GLK (also named MAP4K3) induces activation of NF-κB, which contributes to tumor progression, we investigated the role of GLK in NSCLC. GLK protein levels of 190 samples from pulmonary tissue arrays and 58 pulmonary resection samples from stage I to stage III NSCLC patients were studied using immunohistochemistry or immunoblotting. High levels of GLK proteins were detected in pulmonary tissues from NSCLC patients. Elevated GLK protein levels were correlated with increased recurrence risks and poor recurrence-free survival rates in NSCLC patients after adjusting for pathologic stage, smoking status, alcohol status, and EGFR levels. Thus, GLK is a novel prognostic biomarker for NSCLC recurrence.

## INTRODUCTION

Lung cancer is the leading cause of cancer death worldwide and accounted for 1.37 million deaths in 2008 (WHO) [1]; such deaths are projected to continue rising [1, 2]. Non-small cell lung cancer (NSCLC), which accounts for about 85% of lung cancer, is divided into three main subtypes: squamous cell carcinoma, adenocarcinoma, and large cell carcinoma. Epidermal growth factor receptor (EGFR) is overexpressed in up to 60% of NSCLCs and is a therapeutic target [3]. Unfortunately, the therapeutic inhibition of EGFR results in cancer regressions in only 10% to 20% of NSCLC patients [4]. In addition, despite optimal surgical treatments of stage I or stage II NSCLCs, about 40% to 60% of patients still die of recurrence [5, 6], which is the appearance of a new episode after therapy. Thus, it is essential to identify novel biomarkers or even therapeutic targets for NSCLC tumorigenesis and recurrence.

Mitogen-activated protein kinase kinase kinase kinase (MAP4K) family — including HPK1/MAP4K1 [7, 8], GCK/MAP4K2, GLK/MAP4K3 [9–12], HGK/MAP4K4 [13–15], and KHS/MAP4K5 — is a subgroup of the mammalian STE20 family of serine/threonine protein kinases [16, 17]. MAP4K family kinases are the upstream regulators of the MAP kinase cascades that regulate various cellular functions, such as proliferation, apoptosis, and migration [18, 19]. HPK1/MAP4K1 is involved in cancer cell transformation and invasion [20–22]. HGK/MAP4K4 controls cancer cell mobility [23, 24], and its expression is correlated with worse prognosis in patients with pancreatic ductal adenocarcinoma [25]. GLK can promote cell growth via activating the mTOR signaling pathway after amino acid treatment in epithelial cell lines. GLK positively regulates NF-κB signaling, leading to the development of autoimmune diseases [10–12, 26]. Activation of the NF-κB signaling pathway is involved in carcinogenesis and in cancer resistance to chemotherapy or radiation therapy [27, 28]. Thus, we studied whether GLK is involved in cancer progression using NSCLC samples. Our first objective was to examine whether GLK protein levels correlate with any clinicopathologic parameters. The second goal was to determine whether GLK protein levels correlate with cancer recurrence in NSCLC patients. The third goal was to study whether GLK protein levels are associated with the recurrence-free period/survival in NSCLC patients (Supplementary Figure S1).

## RESULTS

### Elevated GLK protein levels in pulmonary tissues from NSCLC patients

GLK protein levels in a pulmonary tissue array containing 158 human NSCLCs, 13 cancer-adjacent tissues, and 19 normal pulmonary tissues were examined using immunohistochemical staining (Supplementary Table S1 and Supplementary Figure S2). GLK proteins were localized mainly in the cytoplasm of pulmonary cells by immunohistochemistry analysis (Supplementary Figure S2). The expression levels of GLK proteins in NSCLCs were higher than those in normal pulmonary tissues (Supplementary Figure S2A and S2B). GLK protein levels were elevated in 91.9% of the NSCLCs but in only 3.1% of the normal pulmonary tissues (Supplementary Table S1A). The pulmonary tissues from 158 NSCLC patients showed relative GLK expression levels averaging 2.00 ± 0.59 (mean ± SD). Thirteen cancer-adjacent pulmonary tissues showed relative GLK expression levels averaging 1.58 ± 0.56 (mean ± SD). In contrast, 19 normal pulmonary tissues showed relative GLK expression levels averaging 0.87 ± 0.39 (Supplementary Table S1A). GLK protein levels were increased in both tumor types (adenocarcinoma and squamous cell carcinoma) and in all pathologic stages of NSCLCs (Supplementary Table S1A), suggesting that GLK is involved in the carcinogenesis of NSCLCs.

### GLK overexpression in NSCLC

To further study the role of GLK in NSCLCs, we analyzed clinical lung tissues from 58 previously untreated NSCLC patients who underwent pulmonary resection. The baseline demographics of the enrolled patients are listed in Table 1. The specimens were from 58 enrolled patients with an average age of 65.0 ± 11.3 (mean ± SD) years old. There were 23 females (39.7%) and 35 males (60.3%), as well as 27 nonsmokers (46.6%) and 31 smokers (53.4%). Thirty-seven patients (63.8%) had adenocarcinoma and 21 patients (36.2%) had squamous cell carcinoma; in this study 44 patients (75.9%) had stage I or II NSCLCs and 14 patients had (24.1%) stage III NSCLCs (Table 1). Twenty-four patients (41.4%) had recurrence 12–24 months after resection, and 34 patients (58.6%) did not. The recurrence site was the lung (new episodes), the bone, the brain, or the liver. At the time of the most recent follow-up, 4 patients were deceased and 54 patients were alive.

**Table 1:** Clinicopathologic correlation of GLK protein levels in NSCLC patients

Subject characteristics	n (%) Median^†^	GLK-Low (n = 33)	GLK-High (n = 25)	*P*^‡^
Age (years)	65.0 (11.3)			
< 60	18 (31.0)	8	10	0.256
≥ 60	40 (69.0)	25	15	
Gender				
Male	35 (60.3)	16	19	0.569
Female	23 (39.7)	17	6	
Surgical procedure				
Limited	6 (10.3)	5	1	0.349
Lobectomy	49 (84.5)	27	22	
Pneumonectomy	3 (5.2)	1	2	
Histologic grade				
Well	5 (8.6)	5	0	0.143
Moderate	27 (46.6)	14	13	
Poor	26 (44.8)	14	12	
Tumor type				
SCC	21 (36.2)	10	10	0.578
AC	37 (63.8)	23	15	
Pathologic stage				
I + II	44 (75.9)	26	18	0.758
III	14 (24.1)	7	7	
T-stage				
T1	8 (13.8)	5	3	1.000
T2	34 (58.6)	19	15	
T3 + T4	16 (27.6)	9	7	
N-stage				
N0	39 (67.2)	22	17	1.000
N1+N2	19 (32.8)	11	8	
M-stage				
M0	57 (98.3)	33	24	0.247
M1	1 (1.7)	0	1	
Smoking status				
Nonsmoker	27 (46.6)	18	9	0.207
Current smoker	14 (24.1)	5	9	
Former smoker	17 (29.3)	10	7	
Alcohol status				
No	49 (84.5)	30	19	0.154
Yes	9 (15.5)	3	6	
Recurrence				
No	34 (58.6)	24	10	0.012*
Yes	24 (41.4)	9	15§	

Abbreviations: NSCLC, non-small cell lung cancer; SCC, squamous cell carcinoma; AC, adenocarcinoma

* *P*-value < 0.05, statistical significance

† Categorical data: n (%); Continuous variables: Mean (SD)

‡ *P* values were calculated with the use of the Fisher's Exact Test

§ Relative risk, 2.20, 95% CI, 1.157–4.183

**Figure 1:**
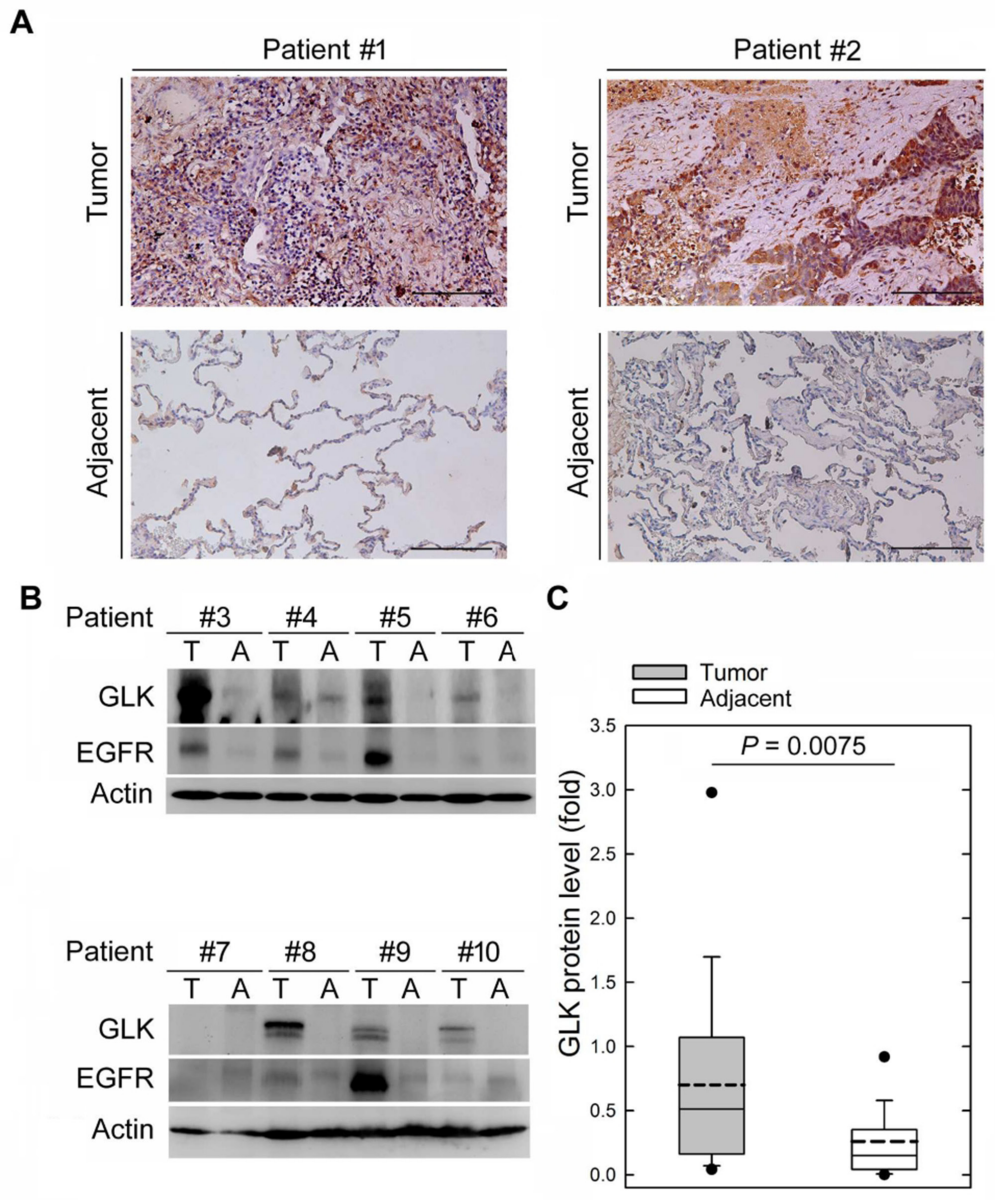
GLK proteins are overexpressed in lung-tumor tissues in NSCLC patients **A.** Immunohistochemical examination of GLK proteins in the NSCLC tissues and the tumor-adjacent tissues from two representative patients. Anti-GLK #1 antibody was used. **B.** Immunoblotting of GLK, EGFR, and actin levels in the NSCLC tissues (T) and the tumor-adjacent tissues (A) from eight representative NSCLC patients. Anti-GLK #1 antibody was used. **C.** Densitometry analyses of the immunoblotting data from 58 human patients with NSCLC. Relative fold changes were normalized to actin. Means ± SD. P = 0.0075 (Student's *t*-test).

Consistent with the tissue-array data, tumor tissues from NSCLC patients also showed GLK overexpression using immunohistochemistry staining (Figure 1A). In contrast, GLK mRNA levels were not changed in NSCLC tissues from searching the USA NCBI Gene Expression Omnibus database (GEO Profile, GDS 3837; Supplementary Figure S3). To better quantify the levels of GLK proteins in NSCLC tissues (T) and paired tumor-adjacent tissues (A), immunoblotting analysis was used for the rest of the studies. GLK protein levels in NSCLC tissues (T) and paired tumor-adjacent tissues (A) were verified using immunoblotting. GLK protein levels in tumor tissues were higher than those in the paired tumor-adjacent tissues from 45 of 58 (78%) patients with NSCLCs (Figure 1B and 1C). Quantitative analyses showed that GLK protein levels in NSCLC tissues (GLK_T_) were significantly higher (0.92 ± 1.83) than those in the paired tumor-adjacent tissues (GLK_A_, 0.26 ± 0.3, *P* = 0.0075; Figure 1C). The relative GLK protein levels in tumor tissues versus those in tumor-adjacent tissues from individual NSCLC patients were calculated (GLK_R_ = GLK_T_/GLK_A_). To study whether GLK protein levels act as a biomarker of NSCLCs, the relative GLK protein levels (GLK_R_) were subjected to the receiver operating characteristic (ROC) curve analysis. The ROC curve showed that when GLK_R_ values were more than 2.93 fold, the area under the curve was 62.4%, the sensitivity was up to 62.5%, and the specificity was up to 70.6%. Patients were divided into GLK-High (> 2.93-fold) and GLK-Low (≤ 2.93-fold) groups according to the ROC cutoff value (2.93) (Table 1). We found no association between GLK protein levels and other clinicopathologic parameters, including pathologic stage, gender, age, histological characteristics, smoking status, and alcohol status (Table 1). Consistently, GLK protein levels also were not associated with these clinicopathologic parameters (lacking recurrence data) that were provided by BioMax, Inc. (Supplementary Table S1B). Notably, GLK-High was significantly associated with NSCLC recurrence (Table 1). Moreover, the GLK-High patients showed a higher risk for recurrence (relative risk, 2.20, 95% confidence interval (CI), 1.157–4.183) than the GLK-Low patients (Table 1), suggesting that GLK overexpression may be a risk factor of recurrence for NSCLC.

Previous studies have shown that the EGFR protein is overexpressed in many NSCLC patients [3, 29]. The EGFR protein levels in NSCLC patients were also calculated (EGFR_R_ = EGFR_Tumor_/ EGFR_Adjacent_). As expected, we found that EGFR protein levels were higher in tumor tissues (1.04 ± 1.38 to 0.32 ± 0.56, *P* = 0.0003; Figure 1A and Supplementary Figure S4). Notably, Pearson correlation coefficient analysis showed that EGFR_R_ values were not correlated with GLK_R_ values (r = 0.04005, *P* = 0.7653), suggesting that GLK overexpression is independent of EGFR upregulation in NSCLC tissues. Next, we studied whether EGFR upregulation is also correlated with NSCLC recurrence. Patients were divided into EGFR-High (> 1.07-fold) and EGFR-Low (≤ 1.07-fold) groups according to the ROC cutoff value (1.07). We found that EGFR-High was not associated with NSCLC recurrence (*P* = 0.74; Supplementary Table S2). Taken together, the results suggest that GLK overexpression, but not EGFR overexpression, is a putative prognostic biomarker for NSCLC recurrence.

### Correlation between GLK protein levels and NSCLC recurrence

Next, we compared the association between estimated risks and NSCLC recurrence in 58 patients, using univariate logistic regression analysis. GLK protein levels were significantly associated with NSCLC recurrence (odds ratio (OR) = 4.00, 95% CI, 1.32–12.11, *P* = 0.014; Supplementary Table S3), while smoking status, alcohol status, and EGFR protein levels were not. Similar to the previous studies [24], the pathologic stage was modestly associated with NSCLC recurrence (OR = 3.48, 95% CI, 0.99–12.25, *P* = 0.052; Supplementary Table S3).

In multivariate logistic regression analysis, NSCLC patients with GLK-High had high risks of cancer recurrence (n = 58, OR = 9.98, 95% CI, 2.24–44.5, *P* = 0.003) after adjusting for the pathologic stage, smoking status, alcohol status, and EGFR protein levels (Table 2). The association between cancer recurrence and GLK overexpression in NSCLC patient occurred in both males (n = 35, OR = 11.66, 95% CI, 1.46–93.02, *P* = 0.021; Table 2) and females (n = 23, OR = 12.28, 95% CI, 0.93–161.80, *P* = 0.057; Table 2). The association between the recurrence risks and GLK protein levels was higher than that between the recurrence risks and the pathologic stages (OR = 3.67, 95% CI, 0.82–16.4, *P* = 0.089; Table 2) in NSCLC patients with a surgery resection.

**Table 2:** Multivariate logistic regression analysis for the estimated risk of recurrence in NSCLC patients

Variable	All (n = 58)		Female (n = 23)		Male (n = 35)	
	Odds Ratio (95% CI)	*P*^†^	Odds Ratio (95% CI)	*P*^†^	Odds Ratio (95% CI)	*P*^†^
GLK (High vs. Low)	9.98 (2.24–44.5)	0.003*	12.28 (0.93–161.80)	0.057	11.66 (1.46–93.02)	0.021*
Pathologic stage	3.67 (0.82–16.4)	0.089	6.15 (0.26–143.48)	0.258	3.32 (0.47–23.50)	0.230
Smoking status						
Current smoker vs. Nonsmoker	0.23 (0.04–1.37)	0.106	NA	—	0.05 (0.00–0.97)	0.048*
Former smoker vs. Nonsmoker	0.32 (0.06–1.77)	0.192	3.45 (0.17–72.10)	0.425	0.05 (0.00–0.90)	0.043*
Alcohol status	1.53 (0.23–9.92)	0.658	NA	—	2.02 (0.28–14.32)	0.483
EGFR	0.93 (0.86–1.02)	0.125	0.90 (0.70–1.15)	0.386	0.92 (0.83–1.02)	0.115

Abbreviations: NSCLC, non-small cell lung cancer

NA: due to the small sample size

* *P*-value < 0.05, statistical significance

† *P* values: multivariate logistic regression analysis

To further study the impact of GLK protein levels on the hazard of recurrence-free lifetime variables, we examined the associations between the clinicopathologic parameters and recurrence-free time periods. According to univariate Cox proportional hazards regression analysis, both GLK protein expression (hazard ratio (HR) = 2.56, 95% CI, 1.12–5.86, *P* = 0.026) and the pathologic stage (HR = 2.30, 95% CI, 1.00–5.28, *P* = 0.050) were associated with recurrence-free survival in NSCLC patients (n = 58, Supplementary Table S4). In multivariate Cox proportional hazards regression analysis, after adjusting for the clinicopathologic parameters, the GLK protein level was an independent prognostic factor for recurrence-free survival (HR = 3.80, 95% CI, 1.59–9.09, *P* = 0.003; Table 3). The association between the recurrence-free survival rates and GLK protein levels was higher than that between the recurrence-free survival rates and the pathologic stages (HR = 2.11, 95% CI, 0.90–4.97, *P* = 0.087; Table 3) in NSCLC patients. Furthermore, there were significant associations between the prediction for recurrence-free survival and the expression of GLK proteins in both females (n = 23) and males (n = 35) with NSCLCs (HR = 4.46, 95% CI, 1.11–17.88, *P* = 0.035 in females; HR = 4.75, 95% CI, 1.32–17.12, *P* = 0.017 in males; Table 3).

**Table 3:** Multivariate Cox proportional hazards regression analysis for the prediction of recurrence-free survival in NSCLC patients

Variable	All (n = 58)		Female (n = 23)		Male (n = 35)	
	Hazard Ratio (95% CI)	*P*^†^	Hazard Ratio (95% CI)	*P*^†^	Hazard Ratio (95% CI)	*P*^†^
GLK (High vs. Low)	3.80 (1.59–9.09)	0.003*	4.46 (1.11–17.88)	0.035*	4.75 (1.32–17.12)	0.017*
Pathologic stage	2.11 (0.90–4.97)	0.087	2.28 (0.48–10.93)	0.303	2.17 (0.71–6.67)	0.176
Smoking status						
Current smoker vs. Nonsmoker	0.47 (0.16–1.40)	0.174	NA	—	0.21 (0.05–0.91)	0.037*
Former smoker vs. Nonsmoker	0.50 (0.16–1.57)	0.237	3.62 (0.65–20.31)	0.144	0.14 (0.03–0.78)	0.025*
Alcohol status	1.78 (0.55–5.77)	0.336	NA	—	2.77 (0.76–10.10)	0.123
EGFR	0.95 (0.88–1.02)	0.156	0.90 (0.74–1.10)	0.319	0.95 (0.88–1.02)	0.171

Abbreviations: NSCLC, non-small cell lung cancer

NA: due to the small sample size

* *P*-value < 0.05, statistical significance

† *P* values: multivariate Cox proportional hazards regression analysis

**Figure 2:**
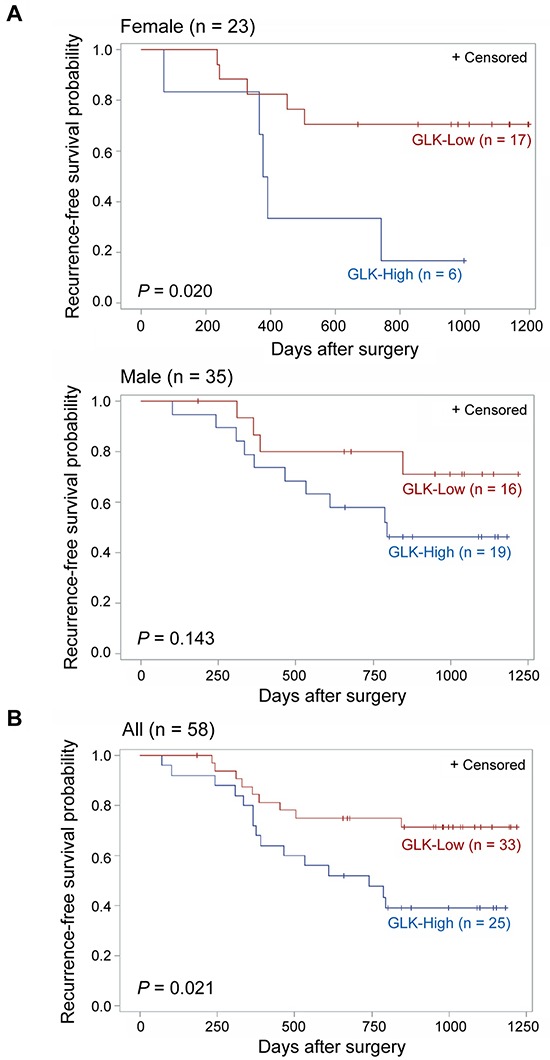
Kaplan-Meier estimates of recurrence-free survival according to GLK protein levels (GLK-High *versus* GLK-Low) of NSCLCs **A.** Grouping by gender. Females (n = 23; upper panel) and males (n = 35; lower panel). **B.** All NSCLC patients (n = 58). *P* values were obtained with the use of a log-rank test. Tick marks indicate patients whose data were censored by the time of most recent follow-up.

In addition, using the Kaplan-Meier survival analysis, NSCLC patients with GLK overexpression showed poor recurrence-free survival in females (n = 23, GLK-High *versus* GLK-Low, *P* = 0.020) but not in males (n = 35, GLK-High *versus* GLK-Low, *P* = 0.143). Nonetheless, poor recurrence-free survival was still shown in all GLK-overexpressed NSCLC patients (n = 58, GLK-High *versus* GLK-Low, *P* = 0.021; Figure 2). As expected, the pathologic stage was also correlated with recurrence-free survival (stage I+II *versus* stage III, *P* = 0.043; Supplementary Figure S5A). According to the tumor types, the reduced time periods of recurrence-free survival were observed in adenocarcinoma patients with GLK-High (n = 38, GLK-High *versus* GLK-Low, *P* = 0.035) but not in squamous cell carcinoma patients with GLK-High (n = 20, GLK-High *versus* GLK-Low, *P* = 0.245; Supplementary Figure S5B). Thus, NSCLC patients with GLK overexpression have a worse prognosis than the patients without GLK overexpression, especially in adenocarcinoma.

## DISCUSSION

NSCLC is an aggressive cancer with poor survival for patients; therefore, it is essential to find the diagnostic and even prognostic biomarkers for more effective cancer diagnosis and treatment. Here we demonstrate that GLK overexpression occurred in NSCLC tissues and was independent of tumor stages. GLK overexpression was significantly correlated with high recurrence risks and poor recurrence-free survival rates. Moreover, the associations of the recurrence risks or the recurrence-free survival rates with GLK protein levels were equivalent to or higher than those with the pathologic stages of NSCLC patients. The association between the pathologic stages and the recurrence risks may be underestimated due to the small sample size of late-stage (only 24% for stage III, none for stage IV) patients. Our findings suggest that the GLK protein is a useful prognostic biomarker for NSCLC recurrence of early-stage (stage I/II) patients at the time of surgical resection.

*EGFR* gene mutations/overexpression in NSCLC patients are associated with adenocarcinomas, female, Asian race, or non-smoker [4]. Our data showed that GLK overexpression is independent of EGFR overexpression and is a risk factor for NSCLC recurrence, regardless of the pathologic stage, smoking behavior, alcohol behavior, and EGFR protein levels. The biological basis, natural history, and therapy response between males and females with NSCLCs are distinct [30, 31]. Our data showed that NSCLC patients with higher GLK protein levels were more commonly men than women. Nevertheless, in both genders, GLK protein levels were associated with cancer recurrence after adjusting for the pathologic stage, smoking behavior, alcohol behavior, and EGFR protein levels. Moreover, GLK overexpression was also correlated with a reduced period of recurrence-free survival. Taken together, our data indicate that if NSCLC patients show GLK overexpression in pulmonary tissues, they are likely to be more susceptible to cancer recurrence and to have worse prognosis.

NSCLC accounts for 85% of lung cancers, and 40%–60% of NSCLC patients die of cancer recurrence after a cancer resection. Here we show that GLK proteins are overexpressed in pulmonary tissues from NSCLC patients. Elevated GLK/MAP4K3 protein levels are correlated with increased recurrence risks and poor recurrence-free survival rates in stage I to stage III NSCLC patients. In summary, GLK may be involved in tumor progression of NSCLC. GLK protein levels serve as a prognostic biomarker for cancer recurrence and have prognostic value for recurrence-free survival. Downregulation of GLK proteins may facilitate the development of novel targeted therapies for NSCLC. Further investigations are required to confirm and expand our finding. In addition, GLK mRNA levels were not increased in pulmonary tissues of 60 NSCLC patients from searching the USA NCBI GEO (Gene Expression Omnibus) database. The enhancement of GLK protein levels in NSCLC tissues may be due to translational or post-translational regulation. Thus, the regulatory mechanisms of GLK overexpression or GLK-mediated tumor progression remain to be investigated in the future.

## MATERIALS AND METHODS

### Study design

We collected primary lung tumor specimens from 58 previously untreated NSCLC patients who underwent pulmonary resection in the Division of Thoracic Surgery at Taichung Veterans General Hospital, Taiwan, between November 2009 and November 2012. All specimens, including NSCLC tissues (T, n = 58) and paired tumor-adjacent tissues (A, n = 58), from stage I to stage III NSCLC patients were taken at the time of surgical resection. Tumor types and stages of individual specimens were determined according to the American Joint Committee on Cancer Cancer Staging Manual. Portions of samples were either kept at −80°C until processed for immunoblotting analysis or freshly fixed with formaldehyde and then embedded with paraffin. All experiments were performed in accordance with the guidelines and protocols approved by the Institutional Review Board, Taichung Veterans General Hospital, Taiwan. Every patient provided written informed consent approved by the hospital's Institutional Review Board (approval number: CF13082). Clinical parameters and cancer recurrence data were collected from chart reviews and confirmed by thoracic surgeons.

Tissue array slides (LC1921) were purchased from BioMax (US BioMax, Inc.). The company provided certified documents that all human lung-tissue samples were collected with informed consent. The pulmonary tissue array contained 159 tumor tissues, 13 cancer-adjacent tissues, and 19 normal pulmonary tissues; it was noted that one tumor tissue was missing from this tissue array. We analyzed the GLK protein levels in 190 pulmonary samples from tissue arrays and in 7 pulmonary resection samples from NSCLC patients.

### Antibodies and reagents

Anti-GLK #1 and #2 antibodies were generated by immunization of rabbits with individual peptides [10]. The specificity of anti-GLK antibodies for Immunoblotting has been demonstrated previously [18]. Anti-EGFR antibody was from Cell Signaling Technology. The rest of the chemicals were purchased from Sigma-Aldrich.

### Immunohistochemistry

The tissue sections were deparaffinized, and then treated for antigen retrieval by incubating the slides in boiling buffer (pH 6.0) at 85°C for 10 min. Nonspecific binding was blocked with 3% H_2_O_2_ for 10 min. Then they were blocked with Immunoblock-Ultra V block for 5 min and reacted with anti-GLK #1 or #2 antibodies (1:500) at 4°C overnight. The subsequent steps were carried out using Ultravision Quanto Detection System (Thermo, TL-060-QHL). For negative controls, the primary antibody was replaced with 2% normal serum. Sections were counterstained with Mayer's hematoxylin, and blinded scoring was independently carried out by two individuals according to the intensity (0, not present; 1, minimal; 2, moderate; 3, strong) of GLK expression.

### Immunoblotting analysis

All samples were prepared with lysis buffer (50 mM Tris (pH 8.0), 150 mM NaCl, 1% Triton X-100, 0.5% deoxycholate, 0.1% SDS, 2 μg/ml leupeptin, 5 μg/ml aprotinin, 1 mM PMSF, 1 mM dithiothreitol, and 1 mM Na_3_VO_4_). The equal amounts of protein lysates were fractionated on SDS-PAGE. The protein bands were then transferred to PVDF membranes. After blocking, these membranes were sequentially probed with primary antibody (1:1,000 for anti-GLK #1 or anti-EGFR) and the peroxidase-conjugated secondary antibody (1:8,000). Antibody reaction was performed using the ECL reagent (Millipore), and the membrane was exposed by BioSpectrum 500 imaging system (UVP). Densitometric analysis of the immunoblotting results was performed using GelPro software (Media Cybernetics). Full immunoblots are shown in Supplementary Figure S6.

### Statistical analysis

The associations between clinicopathologic parameters (such as age, gender, smoking status, tumor type, and the pathologic stage) and GLK protein levels were evaluated using the Fisher's exact test. Univariate logistic regression analysis was performed to identify the potential risk factors related to the presence or absence of cancer recurrence. Hazard ratios and corresponding 95% confidence intervals (CI) of recurrence-free periods were calculated using univariate Cox proportional hazards regression model. Variables with *P*-values less than 0.1 in the univariate analysis and those variables of interest were considered to be potentially associated with cancer recurrence / recurrence-free survival [32]. These potential risk factors (variables) were further explored through multivariate analyses. Kaplan-Meier survival analyses were performed to compare the difference in recurrence-free survival between subgroups (e.g., GLK-High *versus* GLK-Low, pathologic stages I + II *versus* pathologic stage III). The log-rank test was used to compare the significance of the survival distributions among two groups. Data were calculated by SAS software, version 9.1 (SAS Institute, Inc., Cary, NC, USA). Two-sided *P*-values less than 0.05 were considered statistically significant. Statistical analyses were performed by a statistician.

## SUPPLEMENTARY FIGURES AND TABLES


