# Supplementary material for: GLK/MAP4K3 overexpression associates with recurrence risk for non-small cell lung cancer
Source: Oncotarget. 2016 May 17;7(27):41748–57. doi: 10.18632/oncotarget.9410 (PMC5173093; doi:10.18632/oncotarget.9410)
Supplement: Supplementary file 1 [file oncotarget-07-41748-s001.pdf]

SUPPLEMENTARY FIGURES AND TABLES

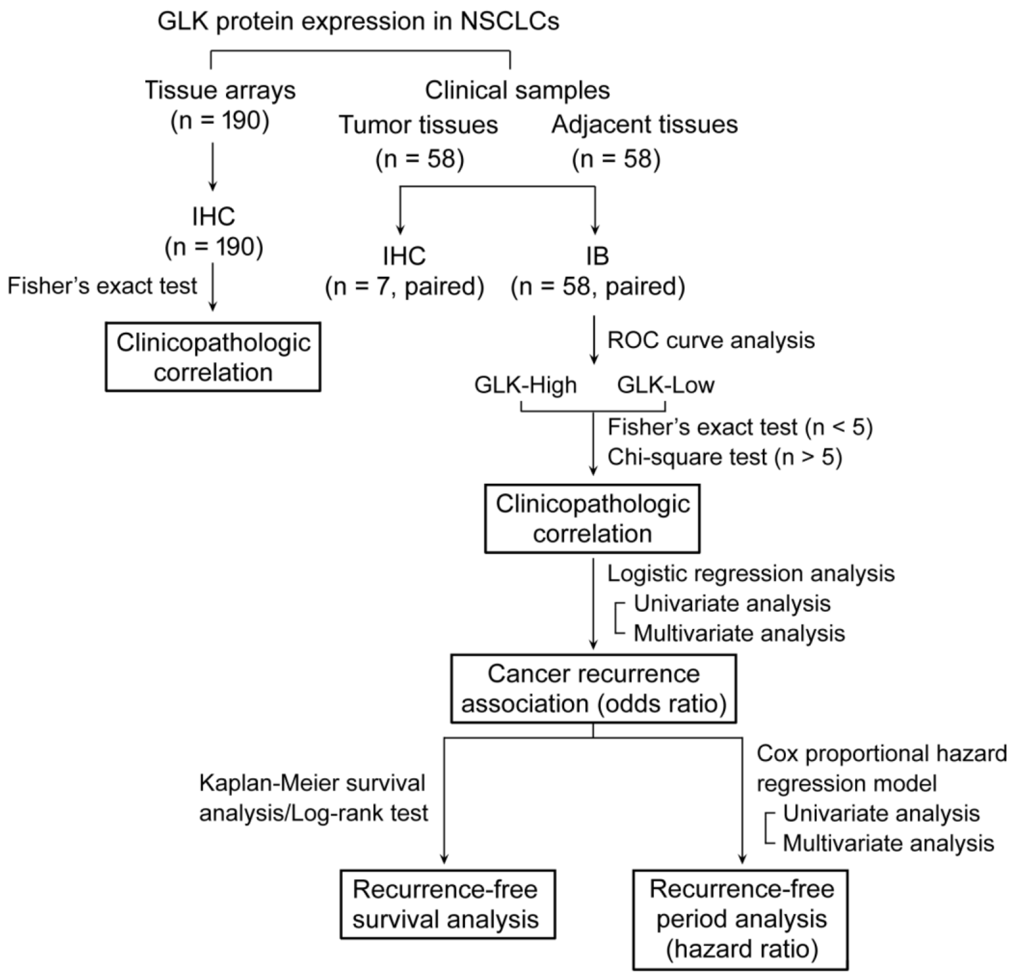

Supplementary Figure S1: Study design of GLK expression analysis in NSCLCs. NSCLCs, non-small cell lung cancers, IHC, immunohistochemistry; IB, immunoblotting.

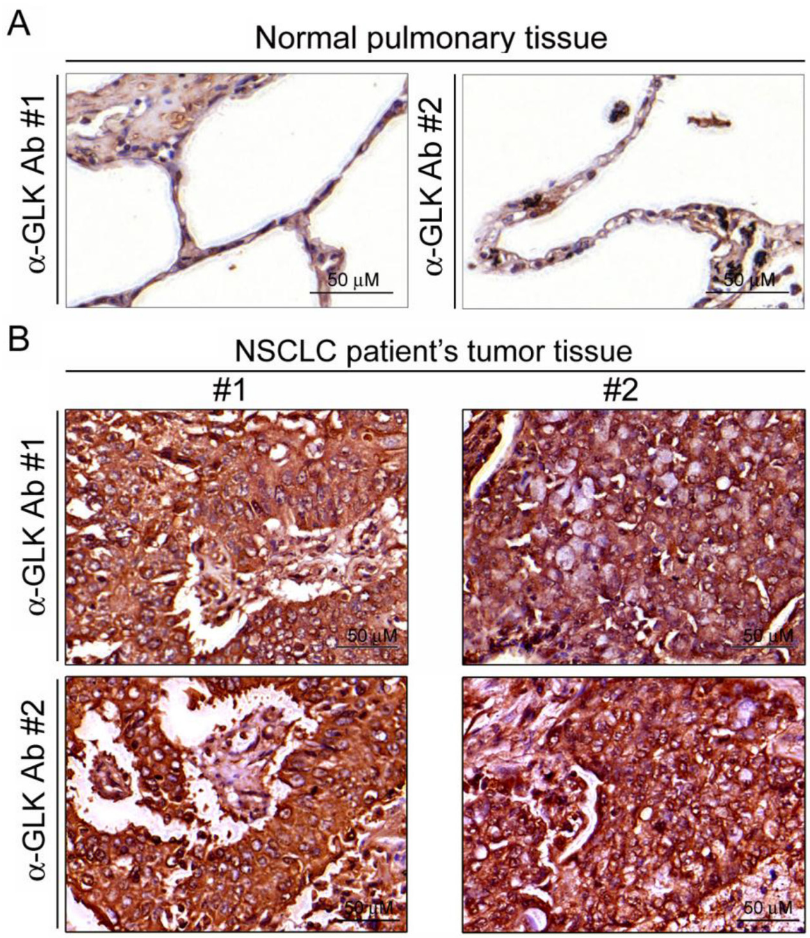

Supplementary Figure S2: Expression of GLK proteins in pulmonary tissues and lung cells. **A.** and **B.** Immunohistochemical examination of GLK protein staining in the normal pulmonary tissues from one normal control (A) and tumor tissues from two representative NSCLC patients (B) using two anti-GLK antibodies (Ab #1 and Ab #2). Same results were obtained using anti-GLK antibodies Ab #1 and Ab #2.

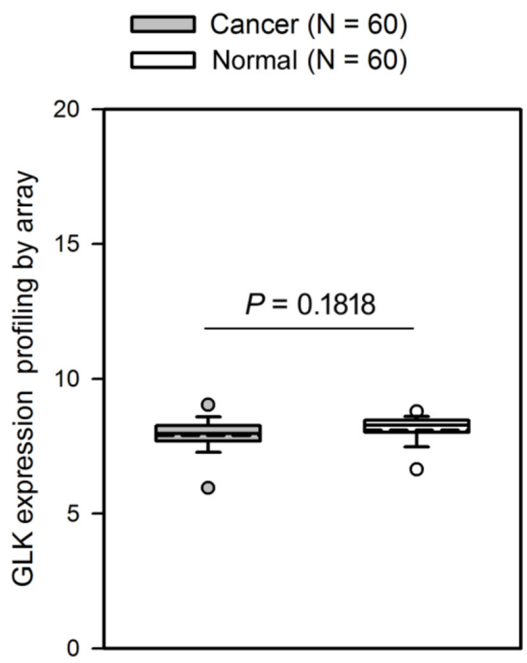

Supplementary Figure S3: GLK mRNA levels are similar in normal and cancer pulmonary tissues. Data were collected from GDS3837 / 218311_at / MAP4K3 (http://www.ncbi.nlm.nih.gov/geo/tools/profileGraph.cgi?ID=GDS3837:218311_at). Means ± SD. $P = 0.1818$ (Student's *t*-test).

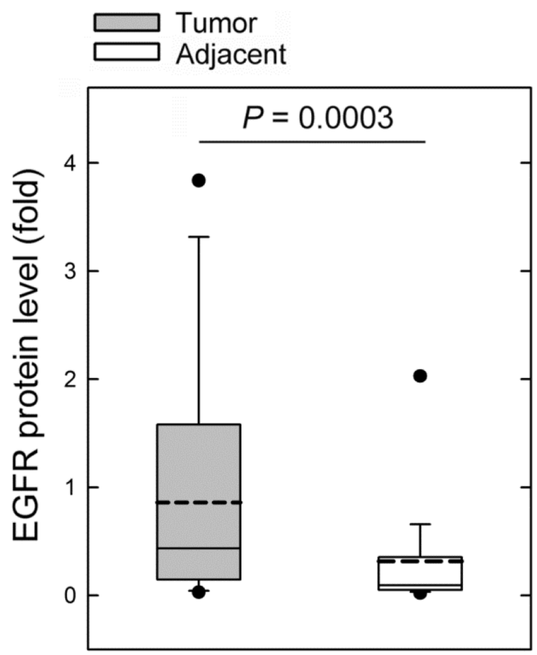

Supplementary Figure S4: EGFR proteins are overexpressed in NSCLC tissues. Densitometry analysis of the immunoblotting data from 58 human NSCLC patients. Relative fold changes were normalized to actin. Means ± SD. $P = 0.0003$ (Student's *t*-test).

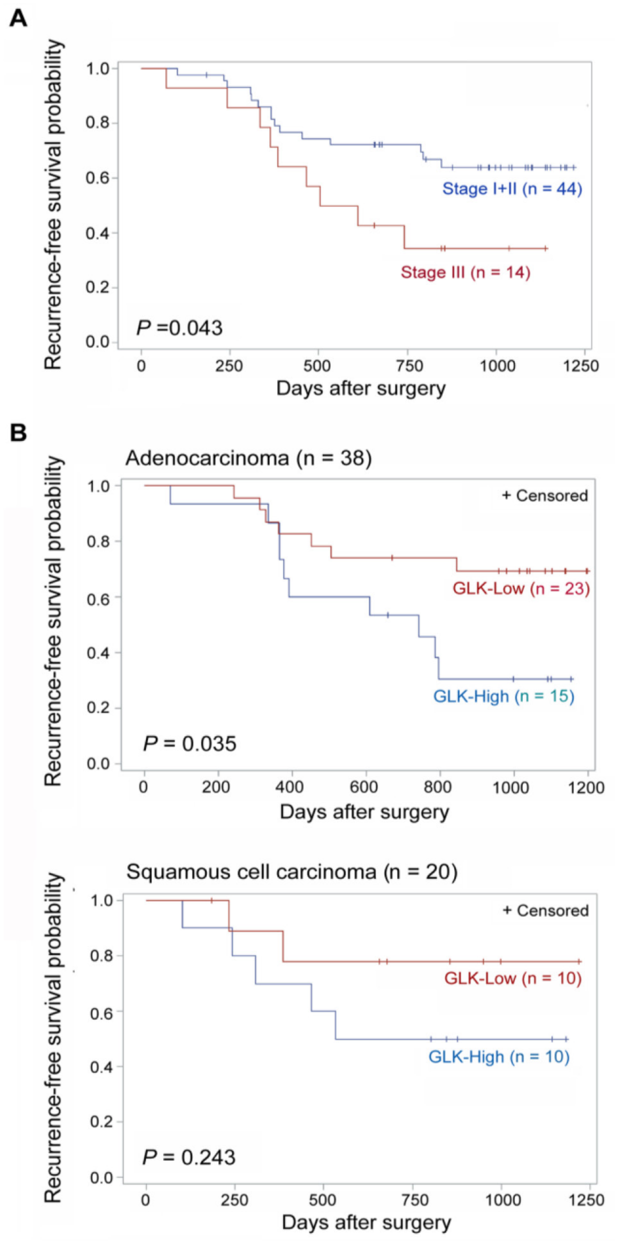

Supplementary Figure S5: Kaplan-Meier curves of recurrence-free survival of NSCLCs. A. Kaplan-Meier curves of recurrence-free survival according to the pathologic stage (I+II *versus* III) of NSCLCs (n = 58). **B**. Kaplan-Meier curves of recurrence-free survival according to the GLK protein levels (GLK-High *versus* GLK-Low) of adenocarcinoma (n = 38; upper panel) or squamous cell carcinoma (n = 20; lower panel). *P* values were obtained with the use of a log-rank test. Tick marks indicate patients whose data were censored by the time of last follow-up.

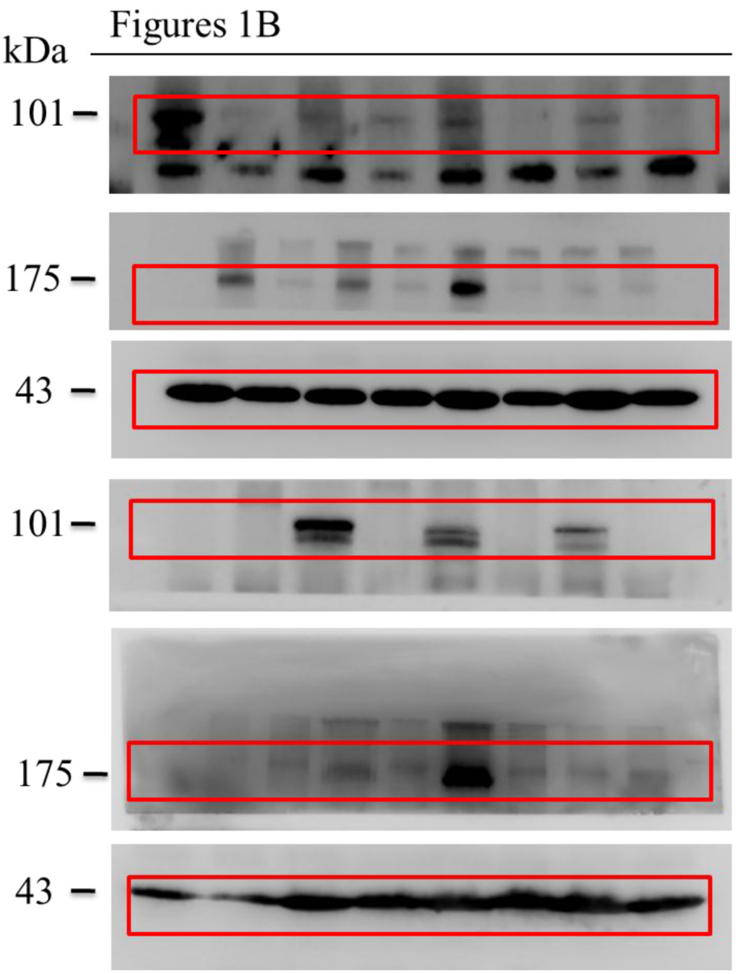

Supplementary Figure S6: Full immunoblots with indicated areas of selection.

Supplementary Table S1: GLK overexpression in the NSCLC tissue array

(A) Immunohistochemistry analysis of GLK levels in the pulmonary tissue array (n = 190; LC1921, BioMax, Inc.)					
**Subject characteristics**	**n (%)**	**Score Mean ± SD**	**Score ≤ 1 (n = 30)**	**Score > 1 (n = 160)**	***P*†**
Normal pulmonary tissue	19 (10.0)	0.87 ± 0.39	14 (46.7)	5 (3.1)	<0.0001*
Cancer adjacent normal pulmonary tissue	13 (6.8)	1.58 ± 0.56	5 (16.7)	8 (5.0)	
Squamous cell carcinoma	80 (42.1)	2.08 ± 0.57	4 (13.3)	76 (47.5)	
Adenocarcinoma	78 (41.1)	1.91 ± 0.61	7 (23.3)	71 (44.4)	

Note: one sample was missing in this tissue array.
* *P*-value < 0.05, statistical significance
†*P* values were calculated with the use of the Fisher's Exact Test.

(B) GLK levels in NSCLCs from the pulmonary tissue array				
**Subject characteristics**	**n (%) Median†**	**Score ≤ 1 (n = 12)**	**Score > 1 (n = 147)**	***P*‡**
Age (years)	56.62 (10.25)			
< 60	88 (55.7)	7	81	0.848
≥ 60	70 (44.3)	5	65	
Gender				
Male	117 (74.1)	6	111	0.080
Female	41 (25.9)	6	35	
Tumor type				
Squamous cell carcinoma	80 (50.3)	4	76	0.221
Adenocarcinoma	79 (49.7)	8	71	
Histologic grade				
Well	15 (10.4)	1	14	1.000
Moderate	89 (61.8)	7	82	
Poor	40 (27.8)	3	37	
T-stage				
T1	13 (8.7)	0	13	0.783
T2	106 (71.1)	10	96	
T3+T4	30 (20.6)	2	28	
N-stage				
N0	59 (43.1)	7	52	0.206
N1+N2+N3	78 (56.9)	4	74	
M-stage				
M0	149 (98.7)	12	137	1.000
M1	2 (1.3)	0	2	

Note: Partial clinicopathologic data of some patients were not available.
†Categorical data: n (%); Continuous variables: Median
‡*P* values were calculated with the use of the Chi-square Test or Fisher's Exact Test.

Supplementary Table S2: Clinicopathologic correlation of EGFR protein levels in NSCLC patients

Characteristics	n (%)	EGFR-Low (n = 12)	EGFR-High(n = 46)	*P*‡
Gender				
Male	35 (60.3)	5	30	0.189
Female	23 (39.7)	7	16	
Pathologic stage				
I + II	44 (75.9)	11	33	0.259
III	14 (24.1)	1	13	
Recurrence				
No	34 (58.6)	8	26	0.744
Yes	24 (41.4)	4	20	

Abbreviations: NSCLC, non-small cell lung cancer
**P*-value < 0.05, statistical significance
‡ *P* values were calculated with the use of the Fisher's Exact Test

Supplementary Table S3: Univariate logistic regression analysis for estimated risk of recurrence in NSCLC patients

Variable	Number of patients (n)	Recurrence rate (%)	Odds Ratio (95% CI)‡	*P*†
Age				
< 60	18	44.4		
≥ 60	40	40.0	0.83 (0.27–2.57)	0.751
Gender				
Male	35	43.5		
Female	23	40.0	0.87 (0.30–2.52)	0.793
Surgical procedure				
Limited	6	33.3		
Lobectomy	49	42.9	1.50 (0.25–8.98)	0.657
Pneumonectomy	3	33.3	1.00 (0.05–18.92)	1.000
Histologic grade				
Well	5	40.0		
Moderate	27	40.7	1.03 (0.15–7.22)	0.976
Poor	26	42.3	1.10 (0.16–7.74)	0.924
Tumor type				
SCC	20	35.0		
AC	38	44.7	1.50 (0.49–4.61)	0.475
Pathologic stage				
I + II	44	34.1		
III	14	64.3	3.48 (0.99–12.25)	0.052

(*Continued*)

Variable	Number of patients (n)	Recurrence rate (%)	Odds Ratio (95% CI)‡	*P*†
T-stage				
T1	8	50.0		
T2	34	35.3	0.55 (0.12–2.58)	0.445
N-stage				
N0	39	35.9		
N1+N2	19	52.6	1.98 (0.65–6.04)	0.228
M-stage				
M0	57	40.4		
M1	1	100.0	NA	—
GLK				
Low	33	27.3		
High	25	60.0	4.00 (1.32–12.11)	0.014*
Smoking status				
Nonsmoker	27	44.4		
Current smoker	14	42.9	0.94 (0.25–3.45)	0.923
Former smoker	17	35.3	0.68 (0.20–2.38)	0.549
Alcohol status				
No	49	38.8		
Yes	9	55.6	1.91 (0.47–8.29)	0.353
EGFR	58	41.4	0.96 (0.90–1.02)	0.227

Abbreviations: NSCLC, non-small cell lung cancer; SCC, squamous cell carcinoma; AC, adenocarcinoma
NA: due to the small sample size
**P*-value < 0.05, statistical significance
†*P* values: univariate logistic regression analysis

Supplementary Table S4: Univariate Cox proportional hazards regression analysis for prediction of recurrence-free survival in NSCLC patients

Variable	Hazard Ratio (95% CI)	*P*†
Age	0.96 (0.41–2.25)	0.927
Gender	0.90 (0.40–2.03)	0.798
Surgical procedure		
Lobectomy vs. Limited	1.02 (0.24–4.36)	0.978
Pneumonectomy vs. Limited	0.72 (0.06–7.92)	0.786
Histologic grade		
Moderate vs. Well	0.88 (0.20–3.99)	0.873
Poor vs. Well	1.04 (0.23–4.69)	0.962
Tumor type	1.13 (0.47–2.72)	0.791
Pathologic stage	2.30 (1.00–5.28)	0.050*
T-stage		
T2 vs. T1	0.66 (0.21–2.05)	0.474
T3 + T4 vs. T1	1.09 (0.33–3.62)	0.890
N-stage	1.70 (0.75–3.83)	0.201
M-stage	6.51 (0.81–52.1)	0.077
GLK (High vs. Low)	2.56 (1.12–5.86)	0.026*
Smoking status		
Current smoker vs. Nonsmoker	1.03 (0.38–2.74)	0.959
Former smoker vs. Nonsmoker	0.82 (0.31–2.18)	0.690
Alcohol	1.82 (0.68–4.89)	0.234
EGFR	0.97 (0.91–1.02)	0.226

Abbreviations: NSCLC, non-small cell lung cancer
* *P*-value < 0.05, statistical significance
†*P* values: univariate Cox proportional hazards regression analysis
